# Supplementary material for: GPCR-induced calcium transients trigger nuclear actin assembly for chromatin dynamics
Source: Nat Commun. 2019 Nov 21;10:5271. doi: 10.1038/s41467-019-13322-y (PMC6872576; doi:10.1038/s41467-019-13322-y)
Supplement: Supplementary file 1 — Supplementary Information [file 41467_2019_13322_MOESM1_ESM.pdf]

Supplementary Information

GPCR-induced calcium transients trigger nuclear actin assembly for chromatin dynamics

Wang et al.

Supplementary Figures

Supplementary Figure 1

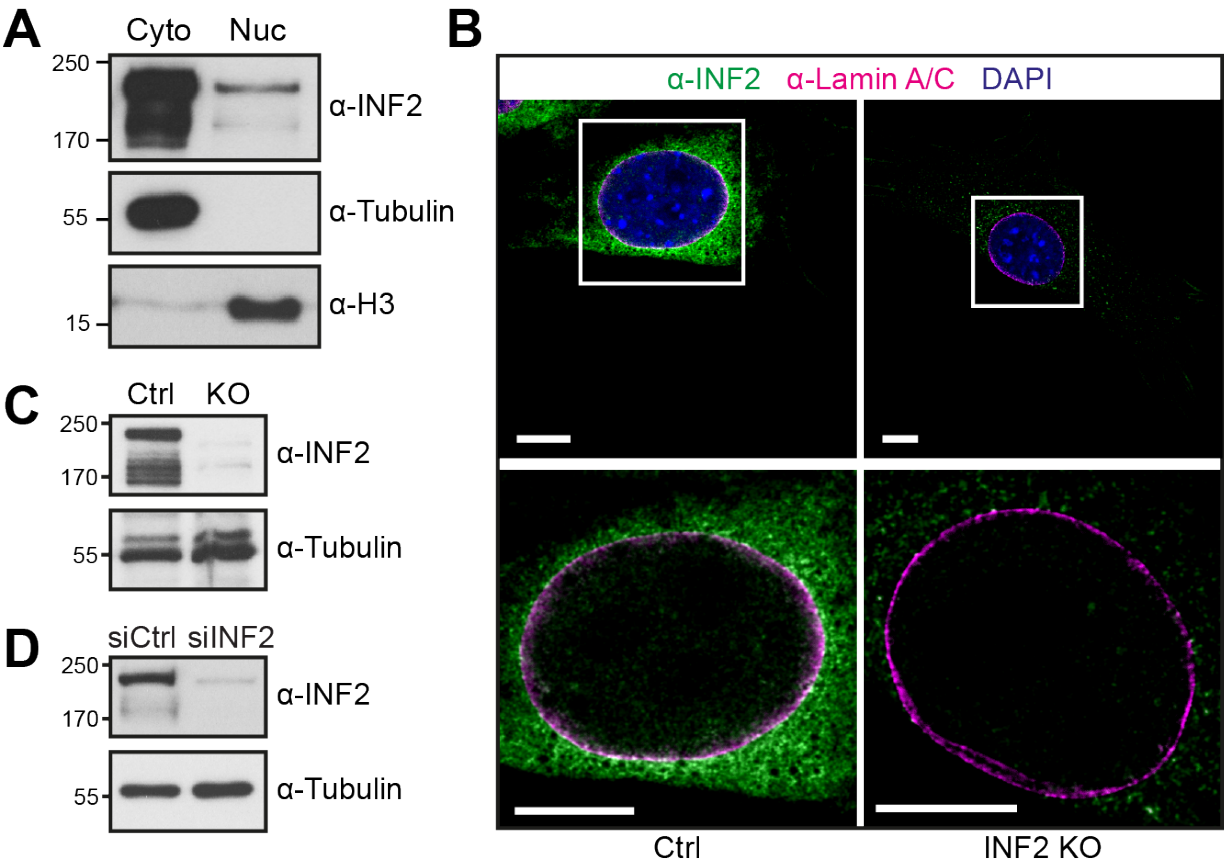

Supplementary Figure1. Endogenous INF2 localization and depletion of INF2 in cells.

A. Fractionation showing INF2 in the cytosol and nucleus. α-Tubulin was used as a cytoplasmic marker. Histone H3 was used as a nuclear marker. **B.** Immunostaining of endogenous INF2 in control or INF2 KO cells showing nuclear localization of INF2 and colocalization of INF2 and Lamin A/C. Representative images out of more than 20 cells for each condition are shown here. Scale bars: 10 μm. **C, D.** Western Blot showing CRISPR/Cas9 (C) or siRNA (D) -mediated INF2 gene depletion.

Supplementary Figure 2

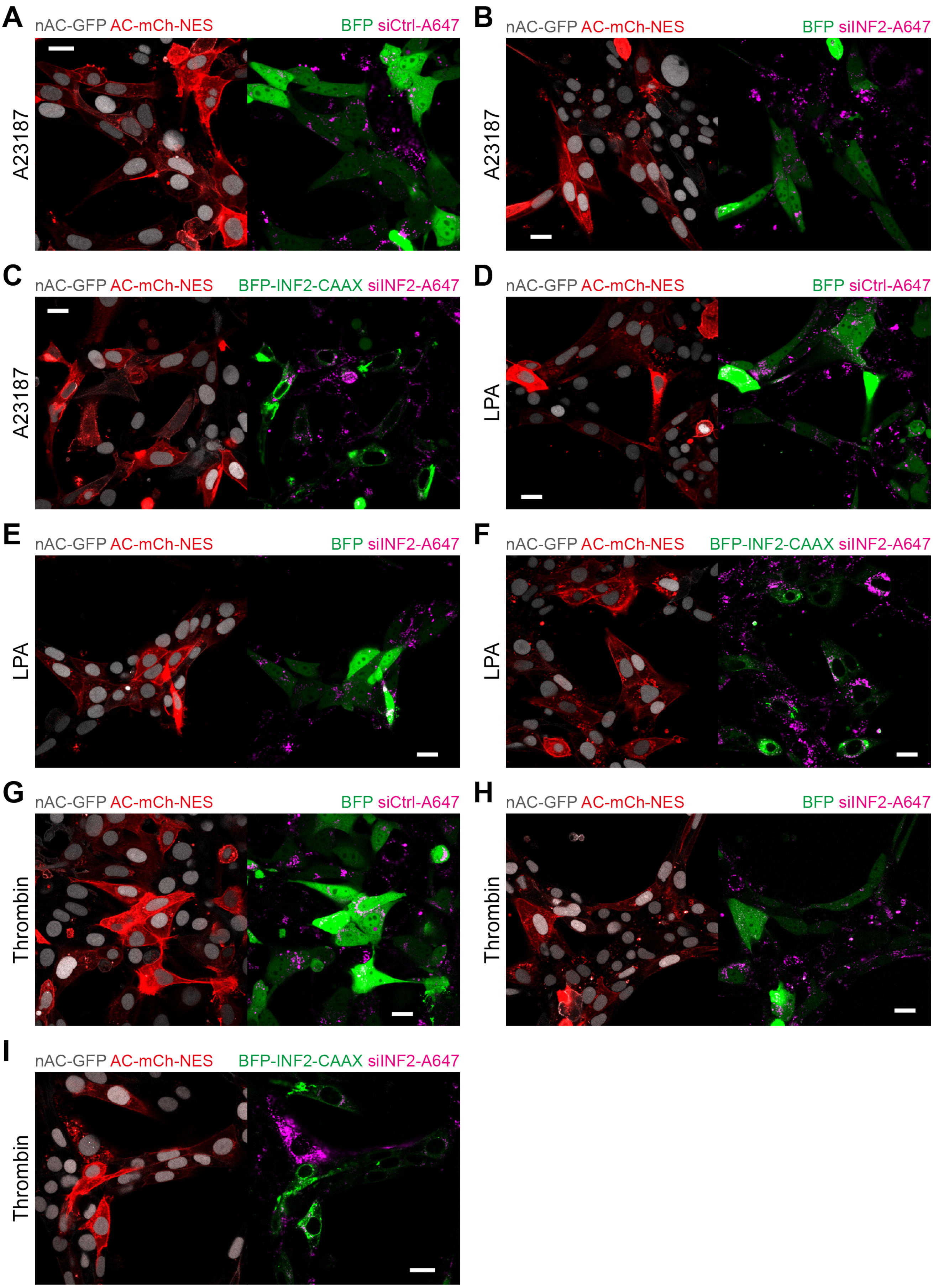

Supplementary Figure 2. Images related to Supplementary Movies 12-20.

A-C. Cells were stimulated with 750 nM A23187. **A.** Image related to Supplementary Movie 12. NIH3T3 cells stably expressing nAC-GFP (gray) were transfected with BFP (green), AC-mCherry-NES (red) and siCtrl-AlexaFluor 647 (magenta). **B.** Image related to Supplementary Movie 13. NIH3T3 cells stably expressing nAC-GFP (gray) were transfected with BFP (green), AC-mCherry-NES (red) and siINF2-AlexaFluor 647 targeting the 3'-UTR (magenta). **C.** Image related to Supplementary Movie 14. NIH3T3 cells stably expressing nAC-GFP (gray) were transfected with BFP-INF2-CAAX (green), AC-mCherry-NES (red) and siINF2-AlexaFluor 647 targeting the 3'-UTR (magenta). **D-F.** Cells were stimulated with 20 μM LPA. **D.** Image related to Supplementary Movie 15. NIH3T3 cells stably expressing nAC-GFP (gray) were transfected with BFP (green), AC-mCherry-NES (red) and siCtrl-AlexaFluor 647 (magenta). **E.** Image related to Supplementary Movie 16. NIH3T3 cells stably expressing nAC-GFP (gray) were transfected with BFP (green), AC-mCherry-NES (red) and siINF2-AlexaFluor 647 targeting the 3'-UTR (magenta). **F.** Image related to Supplementary Movie 17. NIH3T3 cells stably expressing nAC-GFP (gray) were transfected with BFP-INF2-CAAX (green), AC-mCherry-NES (red) and siINF2-AlexaFluor 647 targeting the 3'-UTR (magenta). **G-I.** Cells were stimulated with 0.2U/mL thrombin. **G.** Image related to Supplementary Movie 18. NIH3T3 cells stably expressing nAC-GFP (gray) were transfected with BFP (green), AC-mCherry-NES (red) and siCtrl-AlexaFluor 647 (magenta). **H.** Image related to Supplementary Movie 19. NIH3T3 cells stably expressing nAC-GFP (gray) were transfected with BFP (green), AC-mCherry-NES (red) and siINF2-AlexaFluor 647 targeting the 3'-UTR (magenta). **I.** Image related to Supplementary Movie 20. NIH3T3 cells stably expressing nAC-GFP (gray) were transfected with BFP-INF2-CAAX (green), AC-mCherry-NES (red) and siINF2-AlexaFluor 647 targeting the 3'-UTR (magenta).

Supplementary Figure 3

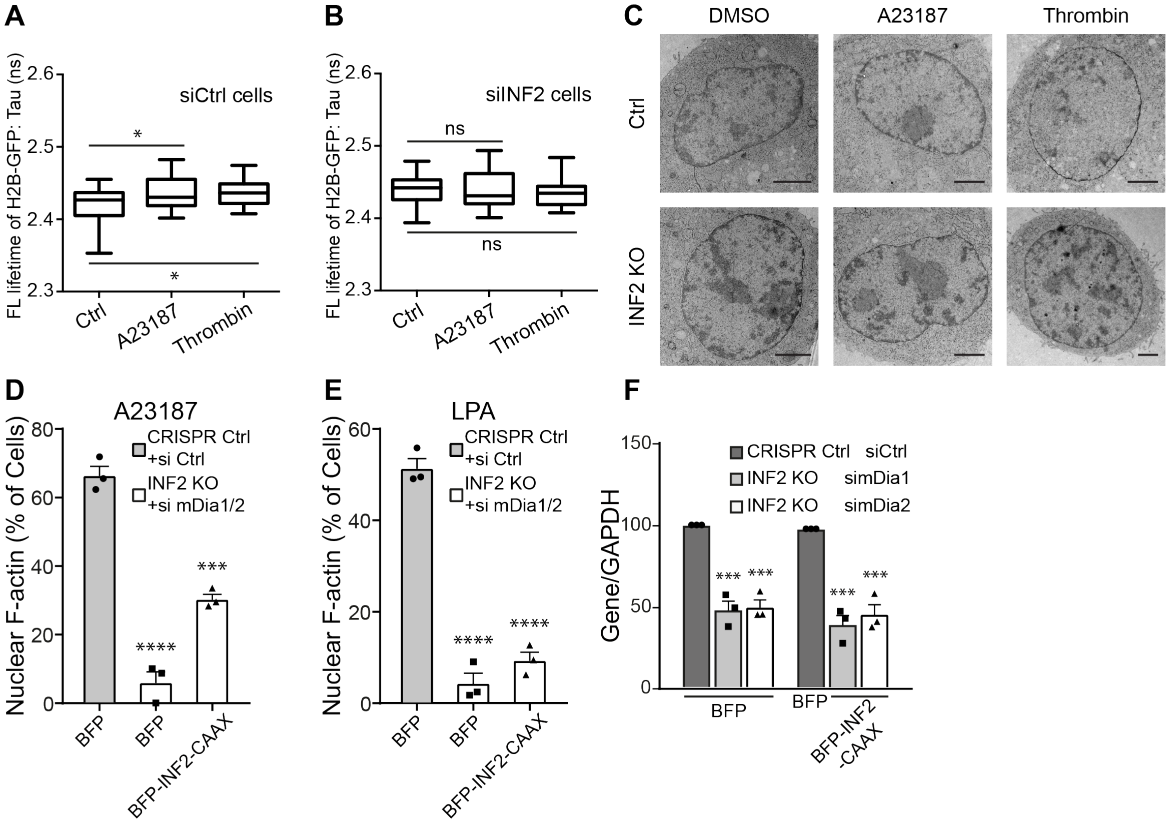

Supplementary Figure 3. INF2 is required for GPCR/Ca^{2+} induced chromatin reorganization and INF2 cooperates with mDia formins in NAA.

A-B. FLIM analysis of cells transfected with control or INF2 siRNA. Cells were treated with DMSO, A23187 or thrombin for 15 min. siCtrl: n=27 (DMSO), 25 (A23187), 27 (thrombin); siINF2: n=26 (DMSO), 26 (A23187), 27 (thrombin) cells. Boxes extend from the 25th to 75th percentiles. Middle line indicates median. Whiskers represent min to max with all points shown. **C.** Representative EM images out of 20 cells for each condition. Scale bars: 2 µm. **D-E.** CRISPR control or INF KO cells stably expressing nAC-GFP were silenced with control or mDia1 and mDia2 siRNA (siCtrl and simDia1 are 3'-AlexaFluor 647 modified). Cells were then transfected with either BFP or BFP-INF2-CAAX. Cells were counted after stimulation with A23187 or LPA. n=3 independent experiments. **F.** qPCR showing mDia1/2 knockdown in G-H. n=3 independent experiments. Error bars: + s.e.m. Source data are provided as a Source Data file.
