# Supplementary material for: GPCR-induced calcium transients trigger nuclear actin assembly for chromatin dynamics
Source: Nat Commun. 2019 Nov 21;10:5271. doi: 10.1038/s41467-019-13322-y (PMC6872576; doi:10.1038/s41467-019-13322-y)
Supplement: Supplementary file 3 — Description of Additional Supplementary Files [file 41467_2019_13322_MOESM3_ESM.pdf]

Description of Additional Supplementary Files

File Name: Supplementary Movie 1

Description: NIH3T3 cells stably expressing nAC-GFP (gray) were transfected with AC-mCherry-NES (red). 750 nM A23187 was added to cells at time point zero. Time-lapse images were recorded showing cytoplasmic actin dynamics and nuclear actin assembly upon the increase of intracellular calcium. Time interval: 4 s. Scale bar: 10 μm.

File Name: Supplementary Movie 2

Description: One single NIH3T3 cell stably expressing nAC-GFP was recorded for nuclear actin assembly upon the treatment of 750 nM A23187. Time interval: 4 s. Scale bar: 10 μm.

File Name: Supplementary Movie 3

Description: NIH3T3 cells stably expressing nAC-GFP (gray) were transfected with AC-mCherry-NES (red). 20 μM LPA was added to cells at time point zero. Time-lapse images were recorded showing cytoplasmic actin dynamics and nuclear actin assembly upon addition of LPA. Time interval: 4 s. Scale bar: 10 μm.

File Name: Supplementary Movie 4

Description: One single NIH3T3 cell stably expressing nAC-GFP was recorded for nuclear actin assembly upon the treatment of 0.2 U/mL thrombin. Time interval: 4 s. Scale bar: 10 μm.

File Name: Supplementary Movie 5

Description: NIH3T3 cells stably expressing nAC-mCherry (gray) were transfected with GCaMP6f (green). 1.5 μM thapsigargin was added to cells at time point zero. Calcium signal and nuclear F-actin were recorded every 2 s. The curves simultaneously show the changes of cyto (green) /nuc (blue) calcium signal and nuclear actin polymerization upon addition of thapsigargin. Scale bar: 10 μm.

File Name: Supplementary Movie 6

Description: Left: the nucleus area is defined as a circle with a diameter of 10 μm. Actin filament formation occurs randomly with an average emergence time of 20 s (Poissonian distributed). The average growth rate of actin filament is set to 1.5 μm/s. Scale bar: 1 μm. Right: Normalized variation (Blue square: fluorescence intensity variance normalized to its range) and the degree of actin polymerization (Red circle, actin monomer in F-actin normalized to its maximum value) were plotted against time in seconds. The quantity of actin monomers within the F-actin (normalized to the peak value when maximum actin filaments form) correlates very well with the fluorescence intensity variance (normalized to its changing range in time).

File Name: Supplementary Movie 7

Description: NIH3T3 cells stably expressing nAC-GFP (green) were transfected with Lamin-CB-mCherry (red) and stimulated by A23187 to visualize nuclear F-actin polymerization. Arrow heads show the tips of actin filaments originating from nuclear envelope. Time interval is 1.5 s. During slow motion movie speed is reduced 5-times. Scale bar: 5 μm.

File Name: Supplementary Movie 8

Description: NIH3T3 CRISPR control cells stably expressing nAC-GFP were transfected with AC-mCherry-NES. 750 nM A23187 was added to cells at time point zero. Time-lapse images were recorded showing cytoplasmic actin dynamics and nuclear actin assembly upon the increase of intracellular calcium. Cells with positive nAC-GFP (gray) and AC-mCherry-NES (red) were counted and quantified. Time interval: 5 s. Scale bar: 20 µm.

File Name: Supplementary Movie 9

Description: NIH3T3 CRISPR INF2 knockout cells stably expressing nAC-GFP were transfected with AC-mCherry-NES. 750 nM A23187 was added to cells at time point zero. Time-lapse images were recorded showing cytoplasmic actin dynamics and nuclear actin assembly upon the increase of intracellular calcium. Cells with positive nAC-GFP (gray) and AC-mCherry-NES (red) were counted and quantified. Time interval: 5 s. Scale bar: 20 µm.

File Name: Supplementary Movie 10

Description: NIH3T3 CRISPR Control cells stably expressing nAC-GFP were transfected with AC-mCherry-NES. 20 µM LPA was added to cells at time point zero. Time-lapse images were recorded showing cytoplasmic actin dynamics and nuclear actin assembly upon the addition of the ligand. Cells with positive nAC-GFP (gray) and AC-mCherry-NES (red) were counted and quantified. Time interval: 5 s. Scale bar: 20 µm.

File Name: Supplementary Movie 11

Description: NIH3T3 CRISPR INF2 knockout cells stably expressing nAC-GFP were transfected with AC-mCherry-NES. 20 µM LPA was added to cells at time point zero. Time-lapse images were recorded showing cytoplasmic actin dynamics and nuclear actin assembly upon the addition of the ligand. Cells with positive nAC-GFP (gray) and AC-mCherry-NES (red) were counted and quantified. Time interval: 5 s. Scale bar: 20 µm.

File Name: Supplementary Movie 12

Description: NIH3T3 cells stably expressing nAC-GFP (gray) were transfected with BFP (green), AC-mCherry-NES (red) and siCtrl-AlexaFluor 647 (magenta). 750 nM A23187 was added to cells at time point zero. Time-lapse images were recorded for only two channels showing cytoplasmic actin dynamics (red) and nuclear actin assembly (gray) upon the increase of intracellular calcium. Cells with positive nAC-GFP, BFP, AC-mCherry-NES and siRNA were counted and quantified. Time interval: 5 s. Scale bar: 20 µm.

File Name: Supplementary Movie 13

Description: NIH3T3 cells stably expressing nAC-GFP (gray) were transfected with BFP (green), AC-mCherry-NES (red) and siINF2-AlexaFluor 647 targeting the 3'-UTR (magenta). 750 nM A23187 was added to cells at time point zero. Time-lapse images were recorded for only two channels showing cytoplasmic actin dynamics (red) and nuclear actin assembly (gray) upon the increase of intracellular calcium. Cells with positive nAC-GFP, BFP, AC-mCherry-NES and siRNA were counted and quantified. Time interval: 5 s. Scale bar: 20 µm.

File Name: Supplementary Movie 14

Description: NIH3T3 cells stably expressing nAC-GFP (gray) were transfected with BFP-INF2-CAAX (green), AC-mCherry-NES (red) and siINF2-AlexaFluor 647 targeting the 3'-UTR (magenta). 750 nM A23187 was added to cells at time point zero. Time-lapse images were recorded for only two channels showing cytoplasmic actin dynamics (red) and nuclear actin assembly (gray) upon the increase of intracellular calcium. Cells with positive nAC-GFP, BFP-INF2, AC-mCherry-NES and siRNA were counted and quantified. Time interval: 5 s. Scale bar: 20 μm.

File Name: Supplementary Movie 15

Description: NIH3T3 cells stably expressing nAC-GFP (gray) were transfected with BFP (green), AC-mCherry-NES (red) and siCtrl-AlexaFluor 647 (magenta). 20 μM LPA was added to cells at time point zero. Time-lapse images were recorded for only two channels showing cytoplasmic actin dynamics (red) and nuclear actin assembly (gray) upon the increase of intracellular calcium. Cells with positive nAC-GFP, BFP, AC-mCherry-NES and siRNA were counted and quantified. Time interval: 5 s. Scale bar: 20 μm.

File Name: Supplementary Movie 16

Description: NIH3T3 cells stably expressing nAC-GFP (gray) were transfected with BFP (green), AC-mCherry-NES (red) and siINF2-AlexaFluor 647 targeting the 3'-UTR (magenta). 20 μM LPA was added to cells at time point zero. Time-lapse images were recorded for only two channels showing cytoplasmic actin dynamics (red) and nuclear actin assembly (gray) upon the increase of intracellular calcium. Cells with positive nAC-GFP, BFP, AC-mCherry-NES and siRNA were counted and quantified. Time interval: 5 s. Scale bar: 20 μm.

File Name: Supplementary Movie 17

Description: NIH3T3 cells stably expressing nAC-GFP (gray) were transfected with BFP-INF2-CAAX (green), AC-mCherry-NES (red) and siINF2-AlexaFluor 647 targeting the 3'-UTR (magenta). 20 μM LPA was added to cells at time point zero. Time-lapse images were recorded for only two channels showing cytoplasmic actin dynamics (red) and nuclear actin assembly (gray) upon the increase of intracellular calcium. Cells with positive nAC-GFP, BFP-INF2, AC-mCherry-NES and siRNA were counted and quantified. Time interval: 5 s. Scale bar: 20 μm.

File Name: Supplementary Movie 18

Description: NIH3T3 cells stably expressing nAC-GFP (gray) were transfected with BFP (green), AC-mCherry-NES (red) and siCtrl-AlexaFluor 647 (magenta). 0.2 U/mL thrombin was added to cells at time point zero. Time-lapse images were recorded for only two channels showing cytoplasmic actin dynamics (red) and nuclear actin assembly (gray) upon the increase of intracellular calcium. Cells with positive nAC-GFP, BFP, AC-mCherry-NES and siRNA were counted and quantified. Time interval: 5 s. Scale bar: 20 μm.

File Name: Supplementary Movie 19

Description: NIH3T3 cells stably expressing nAC-GFP (gray) were transfected with BFP (green), AC-mCherry-NES (red) and siINF2-AlexaFluor 647 targeting the 3'-UTR (magenta). 0.2 U/mL thrombin was added to cells at time point zero. Time-lapse images were recorded for only two channels showing cytoplasmic actin dynamics (red) and nuclear actin assembly (gray) upon the increase of intracellular calcium. Cells with positive nAC-GFP, BFP, AC-mCherry-NES and siRNA were counted and quantified. Time interval: 5 s. Scale bar: 20 μm.

File Name: Supplementary Movie 20

Description: NIH3T3 cells stably expressing nAC-GFP (gray) were transfected with BFP-INF2-CAAX (green), AC-mCherry-NES (red) and siINF2-AlexaFluor 647 targeting the 3'-UTR (magenta). 0.2 U/mL thrombin was added to cells at time point zero. Time-lapse images were recorded for only two channels showing cytoplasmic actin dynamics (red) and nuclear actin assembly (gray) upon the increase of intracellular calcium. Cells with positive nAC-GFP, BFP-INF2, AC-mCherry-NES and siRNA were counted and quantified. Time interval: 5 s. Scale bar: 20 μm.
