# Supplementary material for: GPCR-induced calcium transients trigger nuclear actin assembly for chromatin dynamics
Source: Nat Commun. 2019 Nov 21;10:5271. doi: 10.1038/s41467-019-13322-y (PMC6872576; doi:10.1038/s41467-019-13322-y)
Supplement: Supplementary file 24 — Reporting Summary [file 41467_2019_13322_MOESM24_ESM.pdf]

nature research

Corresponding author(s): Ying Wang and Robert Grosse

Last updated by author(s): Oct 8, 2019

Reporting Summary

Nature Research wishes to improve the reproducibility of the work that we publish. This form provides structure for consistency and transparency in reporting. For further information on Nature Research policies, see Authors & Referees and the Editorial Policy Checklist.

Statistics

For all statistical analyses, confirm that the following items are present in the figure legend, table legend, main text, or Methods section.

n/a	Confirmed	
☐	☒	The exact sample size (*n*) for each experimental group/condition, given as a discrete number and unit of measurement
☐	☒	A statement on whether measurements were taken from distinct samples or whether the same sample was measured repeatedly
☐	☒	The statistical test(s) used AND whether they are one- or two-sided<br>*Only common tests should be described solely by name; describe more complex techniques in the Methods section.*
☒	☐	A description of all covariates tested
☒	☐	A description of any assumptions or corrections, such as tests of normality and adjustment for multiple comparisons
☐	☒	A full description of the statistical parameters including central tendency (e.g. means) or other basic estimates (e.g. regression coefficient) AND variation (e.g. standard deviation) or associated estimates of uncertainty (e.g. confidence intervals)
☐	☒	For null hypothesis testing, the test statistic (e.g. *F*, *t*, *r*) with confidence intervals, effect sizes, degrees of freedom and *P* value noted<br>*Give P values as exact values whenever suitable.*
☒	☐	For Bayesian analysis, information on the choice of priors and Markov chain Monte Carlo settings
☒	☐	For hierarchical and complex designs, identification of the appropriate level for tests and full reporting of outcomes
☒	☐	Estimates of effect sizes (e.g. Cohen's *d*, Pearson's *r*), indicating how they were calculated

Our web collection on statistics for biologists contains articles on many of the points above.

Software and code

Policy information about availability of computer code

Data collection	Quantitative PCR data were collected with Bio-Rad CFX system. Live cell imaging data were collected using Zeiss LSM 800 system or Visitron systems. FLIM data were collected on Leica TCS SP8 system. EM data were acquired on FEI Tecnai 12 TEM.
Data analysis	Statistical data analysis was performed using GraphPad Prism 7 or Microsoft Office Excel. Two-sided Student's t-test was performed when there were only 2 experimental conditions. One-way ANOVA was performed for experiments with more than two conditions. Stars in the figures indicate P values: *: p≤0.05; **: p≤0.01; ***: p≤0.001; ****: p≤0.0001. Images were analyzed with FIJI/Image J (NIH), Zen software (Zeiss) or Metamorph software (Molecular Devices). FLIM data were fitted using the FLIMfit (Imperial College London). For quantification of heterochromatin, nuclei and nucleoli were manually segmented. A FIJI machine learning plugin (Trainable Weka Segmentation) and a macro were used to quantify the region classed as heterochromatin, including the proportion of the nucleus occupied by heterochromatin. Calcium images by GCaMP6f and nAC-mCherry were analyzed by MATLAB. Calcium elevation in the whole cell or nucleus was measured by quantifying the fluorescence intensity of GCaMP6f, and the nuclear actin polymerization was measured by calculating the maximal spatial signal heterogeneity of nAC-mCherry.

For manuscripts utilizing custom algorithms or software that are central to the research but not yet described in published literature, software must be made available to editors/reviewers. We strongly encourage code deposition in a community repository (e.g. GitHub). See the Nature Research guidelines for submitting code & software for further information.

Data

Policy information about availability of data

All manuscripts must include a data availability statement. This statement should provide the following information, where applicable:

- Accession codes, unique identifiers, or web links for publicly available datasets
- A list of figures that have associated raw data
- A description of any restrictions on data availability

The data that support the findings of this work are available from the corresponding authors upon reasonable request. Source data for Fig. 1B-D; Fig. 2A-C; Fig. 3C-

G; Fig. 4A-D; Fig. 5B, D, E-H; Supplementary Figure 1A, C, D; Supplementary Figure 2A, B, D-F are provided in Source Data file.

Field-specific reporting

Please select the one below that is the best fit for your research. If you are not sure, read the appropriate sections before making your selection.

☒ Life sciences ☐ Behavioural & social sciences ☐ Ecological, evolutionary & environmental sciences

For a reference copy of the document with all sections, see nature.com/documents/nr-reporting-summary-flat.pdf

Life sciences study design

All studies must disclose on these points even when the disclosure is negative.

Sample size

For each experiment, sample sizes were decided based on initial testing experiments. Similar experiments published previously were used as references to determine the sample sizes.

Data exclusions

There were no data exclusions in this study.

Replication

All attempts of replication were successful.
In Fig 1B-D, Fig 2A and C, Fig 3C-G, Fig 4D, Supplementary Figure 2D and E, each experiment includes 3 samples in parallel and about 30 cells were recorded and counted each time. Experiments were then repeated 3 or 4 times.
Fig 1A and E show representative images of A23187 or thrombin induced NAA, which were quantified by counting the incidence of cells showing nuclear actin filaments in the main text and in Fig 1D.
Fig 2B and Supplementary Figure 2F are quantitative PCR results from 3 independent experiments with an exception of Galpha13 with only 2 independent experiments.
Fig 2D provides the representative images showing the increase of calcium transients and the NAA upon addition of thapsigargin. Fig 2E provide the quantitative analysis of data from Fig 2D over time and a similar measurement when cells were treated with A23187. The images were analyzed using MATLAB by measuring the fluorescence intensity of GCaMP6f and the maximal spatial signal heterogeneity of nAC-mCherry. In general, the phenomenon observed can be repeated regularly, showing the increase of calcium signal always preceding the appearance of nuclear F-actin. However, due to the operation time of drug stimulation, the extremely rapid response of cells to intracellular calcium increase and the high variability of the fluorescence intensity of GCaMP6f after stimulation, it is difficult to give an average curve with error bars from several different measurements. Therefore, the curves shown in Fig 2E are from only one representative cell analysis respectively.
In Fig 3A is chosen out of 18 cells in multiple independent experiments showing similar observations. Fig 3B is from 15 movies showing similar observations.
Fig 4D gives representative images which were quantified at the same time.
Fig 5A and C provide example images which were further quantified in Fig 5B and D.
Fig 5B, D, G, H and Supplementary Figure 2A, B were measured for GFP fluorescence lifetime.
Western blots were performed 3 times. One representative result is shown. Original uncropped blots are now provided in Source data file.
Supplementary Figure 1B shows the example images of INF2 immunostaining for control or knockout cell lines out of more than 20 cells for each condition.
Supplementary Figure 2C shows representative EM images which were quantified in Fig 5E and F.

Randomization

No randomization was used in the course of the experiments.

Blinding

No blinding was used in this study.

Reporting for specific materials, systems and methods

We require information from authors about some types of materials, experimental systems and methods used in many studies. Here, indicate whether each material, system or method listed is relevant to your study. If you are not sure if a list item applies to your research, read the appropriate section before selecting a response.

Materials & experimental systems

n/a	Involved in the study	
☐	☒	Antibodies
☐	☒	Eukaryotic cell lines
☒	☐	Palaeontology
☒	☐	Animals and other organisms
☒	☐	Human research participants
☒	☐	Clinical data

Methods

n/a	Involved in the study	
☒	☐	ChIP-seq
☒	☐	Flow cytometry
☒	☐	MRI-based neuroimaging

Antibodies

Antibodies used

Antibody name (company, clone/catalog #, dilution)

INF2 (Proteintech, 20466-1-AP, 1:1000 or 1:200)
Gα12 (Santa Cruz, SC-409, 1:500)
Gαq (Santa Cruz, SC-393, 1:1000)
α-Tubulin (Cell Signaling Technology, 11H10, 1:5000)
Histone H3 (Cell Signaling Technology, D1H1, 1:2000)
Flag conjugated with HRP (Sigma, M2, 1:5000)
Flag-conjugated agarose beads (Sigma, M2)
myc conjugated with HRP (Sigma, A5598, 1:2000)
GFP (Santa Cruz, B2, 1:2000)
Lamin A/C antibody (Cell Signaling Technology, 4C11, 1:200)
AlexaFluor 488 donkey anti rabbit IgG (Invitrogen, A21206, 1:200)
AlexaFluor 555 goat anti mouse IgG (Invitrogen, A21424, 1:200)
IgG rabbit-HRP (Bio-Rad, 170-6515, 1:5000)
IgG mouse-HRP (GE Healthcare, 8310, 1:5000)

Validation

Antibodies were purchased from reputable companies as listed below and pre-validated for the following uses.
INF2 (Proteintech, 20466-1-AP): Western blotting after fractionation or in gene knockout experiments, immunostaining showing INF2 subcellular localization.
Gα12 (Santa Cruz, SC-393, 1:1000): Western blotting in gene knockdown experiments.
Gαq (Santa Cruz, SC-409, 1:500): Western blotting in gene knockdown experiments.
α-Tubulin (Cell Signaling Technology, 11H10): Western blotting after fractionation or in gene knockout experiments.
Histone H3 (Cell Signaling Technology, D1H1): Western blotting after fractionation.
Flag conjugated with HRP (Sigma, M2): Western blotting after immunoprecipitation.
Flag conjugated agarose beads (Sigma, M2): immunoprecipitation.
myc conjugated with HRP (Sigma, A5598): Western blotting after immunoprecipitation.
GFP (Santa Cruz, B2): Western blotting after immunoprecipitation.
Lamin A/C antibody (Cell Signaling Technology, 4C11): immunostaining showing nuclear envelope.
AlexaFluor 488 donkey anti rabbit IgG (Invitrogen, A21206): immunostaining.
AlexaFluor 555 goat anti mouse IgG (Invitrogen, A21424): immunostaining.
IgG rabbit-HRP (Bio-Rad, 170-6515): Western blotting.
IgG mouse-HRP (GE Healthcare, 8310): Western blotting.

Eukaryotic cell lines

Policy information about cell lines

Cell line source(s)

Cell lines were originally from ATCC.

Authentication

Cell lines were not authenticated on a molecular level.

Mycoplasma contamination

Cell lines were regularly tested and controlled for mycoplasma contamination throughout this study.

Commonly misidentified lines
(See ICLAC register)

No cell lines used are listed in the ICLAC database.
